# Cross-Sectional Associations between Homoarginine, Intermediate Phenotypes, and Atrial Fibrillation in the Community—The Gutenberg Health Study

**DOI:** 10.3390/biom8030086

**Published:** 2018-08-30

**Authors:** Christoph Niekamp, Dorothee Atzler, Francisco M. Ojeda, Christoph R. Sinning, Karl J. Lackner, Rainer H Böger, Thomas Munzel, Manfred E. Beutel, Irene Schmidtmann, Norbert Pfeiffer, Anja Leuschner, Stefan Blankenberg, Philipp S. Wild, Tanja Zeller, Edzard Schwedhelm, Renate B. Schnabel

**Affiliations:** 1 Department of General and Interventional Cardiology, University Heart Center Hamburg-Eppendorf, 20251 Hamburg, Germany; christophniekamp@gmx.de (C.N.); f.ojeda-echevarria@uke.de (F.M.O.); c.sinning@uke.de (C.R.S.); s.blankenberg@uke.de (S.B.); t.zeller@uke.de (T.Z.); 2 Deutsches Zentrum für Herz-Kreislauf-Forschung e.V. (DZHK), Partner Site Munich, 80336 Munich, Germany; dorothee.atzler@med.uni-muenchen.de; 3 Institute for Cardiovascular Prevention, LMU Munich and Walther-Straub-Institute of Pharmacology and Toxicology, LMU Munich, 80336 Munich, Germany; 4 Center for Cardiovascular Research (DZHK), Partner Site Rhein/Main, 55131 Mainz, Germany; Karl.Lackner@unimedizin-mainz.de (K.J.L.); tmuenzel@uni-mainz.de (T.M.); Philipp.Wild@unimedizin-mainz.de (P.S.W.); schwedhelm@uke.de (E.S.); 5 Institute of Clinical Chemistry and Laboratory Medicine, University Medical Center of the Johannes Gutenberg-University Mainz, 55131 Mainz, Germany; 6 Department of Clinical Pharmacology and Toxicology, University Medical Center Hamburg-Eppendorf, Hamburg, 20246 Hamburg, Germany; boeger@uke.de; 7 Deutsches Zentrum fuer Herz-Kreislauf-Forschung e.V. (DZHK), Partner Site Hamburg/Kiel/Lübeck, 23538 Lübeck, Germany; 8 Center for Cardiology—Cardiology I, University Medical Center of the Johannes Gutenberg-University Mainz, 55131 Mainz, Germany; Anja.Leuschner@unimedizin-mainz.de; 9 Dept Psychosomat Med & Psychotherapy, University Medical Center of the Johannes Gutenberg-University Mainz, 55131 Mainz, Germany; Manfred.Beutel@unimedizin-mainz.de; 10 Institute for Medical Biostatistics, Epidemiology and Informatics, University Medical Center of the Johannes Gutenberg-University Mainz, 55131 Mainz, Germany; Irene.Schmidtmann@unimedizin-mainz.de; 11 Dept Ophthalmology, University Medical Center of the Johannes Gutenberg-University Mainz, 55131 Mainz, Germany; norbert.pfeiffer@unimedizin-mainz.de; 12 Preventive Cardiology and Preventive Medicine, Center for Cardiology, University Medical Center of the Johannes Gutenberg-University Mainz, 55131 Mainz, Germany; 13 Center for Thrombosis and Hemostasis; University Medical Center of the Johannes Gutenberg-University Mainz, 55131 Mainz, Germany

**Keywords:** atrial fibrillation, biomarker, homoarginine, population-based cohort, diastolic disfunction

## Abstract

Homoarginine has come into the focus of interest as a biomarker for cardiovascular disease. Atrial fibrillation (AF) causes a substantial increase in morbidity and mortality. Whether circulating homoarginine is associated with occurrence or persistence of AF and may serve as a new predictive biomarker remains unknown. We measured plasma levels of homoarginine in the population-based Gutenberg health study (3761 patients included, of them 51.7% males), mean age 55.6 ± 10.9 years-old. Associations between homoarginine and intermediate electrocardiographic and echocardiographic phenotypes and manifest AF were examined. Patients with AF (124 patients, of them 73.4% males) had a mean age 64.8 ± 8.6 years-old compared to a mean age of 55.3 ± 10.9 in the population without AF (*p*-value < 0.001) and showed a less beneficial risk factor profile. The median homoarginine levels in individuals with and without AF were 1.9 μmol/L (interquartile range (IQR) 1.5–2.5) and 2.0 μmol/L (IQR 1.5–2.5), respectively, *p* = 0.56. In multivariable-adjusted regression analyses homoarginine was not statistically significantly related to electrocardiographic variables. Among echocardiographic variables beta per standard deviation increase was −0.12 (95% confidence interval (CI) −0.23–(−0.02); *p* = 0.024) for left atrial area and −0.01 (95% CI −0.02–(−0.003); *p* = 0.013) for E/A ratio. The odds ratio between homoarginine and AF was 0.91 (95% CI 0.70–1.16; *p* = 0.45). In our large, population-based cross-sectional study, we did not find statistically significant correlations between lower homoarginine levels and occurrence or persistence of AF or most standard electrocardiographic phenotypes, but some moderate inverse associations with echocardiographic left atrial size and E/A. Homoarginine may not represent a strong biomarker to identify individuals at increased risk for AF. Further investigations will be needed to elucidate the role of homoarginine and cardiac function.

## 1. Introduction

Atrial fibrillation (AF) is the most frequently diagnosed arrhythmia. Its prevalence is increasing due to a variety of suggested reasons such as the aging of the population, more intensive screening, and improved treatment of cardiovascular diseases which lead to a longer survival with the condition [1,2,3]. Lifetime risk of AF after reaching the 4th decade is about 25% [4]. Serious complications of AF are thromboembolic events and development of heart failure [5,6]. Onset of AF is strongly related to higher morbidity and mortality, even after accounting for comorbidities [7,8]. Exploration of new biomarkers is an opportunity to better understand the disease process and possibly identify individuals at increased risk for AF [9].

Although the exact mechanisms are not fully understood, the pathogenesis of AF seems to be linked to oxidative stress and the nitric oxide (NO) pathway [10,11,12]. These observations are in line with the measurement of low NO plasma levels in patients with persistent AF, which returned to normal after electrical cardioversion [13,14].

Homoarginine is a nonproteinogenic amino acid, potentially involved in the NO pathway [15], e.g., by acting as a weak substrate for the NO synthase, which generates NO of L-arginine [16]. It has come into the focus of interest as a biomarker for cardio- and cerebrovascular events [17,18,19,20]. In experimental studies it has been shown that homoarginine exhibits antihypertensive and antithrombotic characteristics [21,22]. It is inversely correlated with subclinical cardiovascular changes and incident disease [16,23,24] and has been suggested to be a novel biomarker for cardiovascular disease risk [18].

The role of homoarginine in AF is less well established. First evidence from an acute coronary syndrome cohort indicates that homoarginine concentrations are lower in individuals with AF [25]. In oesophagectomy patients, lower homoarginine was associated with postoperative AF [26]. These findings suggest a potential role of homoarginine in the pathogenesis of AF.

The lack of knowledge on the role of homoarginine in AF spurred our investigation in the population-based Gutenberg health study, aiming to examine the association between homoarginine levels, the prevalence of AF, electrocardiographic and echocardiographic indicators, reflecting functional and structural cardiac changes in patients with this arrhythmia.

## 2. Materials and Methods

### 2.1. Study Participants

The present data are on the first 5000 consecutive individuals from a total number of 15,000 participants enrolled in the Gutenberg health study. The cohort comprises a population-based, randomly selected sample of the area Mainz/Mainz-Bingen. The recruitment started in 2007 at the Department of Medicine 2, University Medical Centre Mainz. Study participants were enrolled within 10-year age strata from 35 to 74 years-old.

During clinical examination, data on cardiovascular risk factors were collected based on anthropometry and a standardized computer-assisted interview. Smoking status was grouped into the subcategories of non-smokers, such as never smokers and former smokers, and current smokers. Hypertension was defined by an average systolic blood pressure of ≥140 mmHg in two measurements and/or a diastolic blood pressure of ≥90 mmHg and/or anti-hypertensive drug treatment. Dyslipidemia was defined as a low-density lipoprotein/high-density lipoprotein cholesterol ratio of >3.5 and/or based on a physician’s diagnosis. Diabetes was diagnosed if the participant had a fasting blood glucose value of ≥126 mg/dL (minimum fasting period: 8 h) or any blood glucose concentration of ≥200 mg/dL measured on site and/or a physician diagnosis of diabetes was reported.

Prevalent coronary heart disease, self-reported myocardial infarction, stroke, and heart failure were by participant history. Heart failure was verified by clinical aspects (New York Heart Association classification, heart failure medications) and echocardiographic recordings (left ventricular ejection fraction <55%).

For each study participant, a 12-lead electrocardiogram (ECG) (GE Cardiosoft^®^, GE Healthcare, Solingen, Germany) was recorded. The ECG device automatically registered the QTc interval and corrected it via the Bazett formula. The diagnosis of AF was based on AF reported in the medical history and/or atrial fibrillation or atrial flutter registered on the study ECG. Atrial fibrillation was evaluated by at least two doctors with cardiology training and experience in ECG interpretation. In difficult cases, an electrophysiological specialist’s opinion was consulted. Thirty-one participants could not be assigned to the AF or control group.

### 2.2. Biomarker Measurement

Plasma L-homoarginine was measured from ethylenediaminetetraacetic acid (EDTA) plasma aliquots stored at −80 °C using electrospray ionization-liquid chromatography-mass spectrometry with a high-throughput mass spectrometric assay. Briefly summarized, by adding 100 µL of internal standard (2.5 µmol/L [^13^C_6_]-homoarginine) disbanded in methanol to 25 µL of EDTA plasma, proteins were precipitated. The aliquots were centrifuged, vaporized, and afterwards translated to their butyl ester derivatives applying butanolic 1N hydrochloric acid (HCl). After repeated centrifugation, the eluates were dried and again dissolved in 100 µL of methanol:water (25:75) containing 0.1% ammonium format. The plates were positioned in a CTC PAL autosampler (CTC Analytics AG, Zwingen, Switzerland)) and 20 µL samples were injected. Further testing was performed with the mass spectrometer system (Varian 1200 MS, Agilent Technologies, Santa Clara, CA, USA). Lower threshold of quantification for homoarginine was set to be 0.01 µmol/L. Intra- and interassay coefficients of variation were ≤7.5%. Creatinine was determined by routine laboratory method.

### 2.3. Statistical Methods

Participant characteristics were described by median values for continuous variables and absolute and relative frequencies for binary variables. Differences in the homoarginine levels between individuals with and without AF were tested using the Mann–Whitney (*U*) test. Kendall rank correlations for homoarginine, electrocardiographic variables (PQ interval, P wave duration, ventricular rate, QRS duration, QTc interval), echocardiographic variables (left atrial area, E/A, E/E’, deceleration time, left ventricular ejection fraction, left ventricular mass) and AF were computed.

The association of homoarginine and the electrocardiographic and echocardiographic variables were examined via median values and linear regression, with homoarginine as regressor. The models were adjusted for (1) age and sex; (2) age, sex, body mass index (BMI), systolic blood pressure, antihypertensive medication, diabetes, active smoking, family history of myocardial infarction, dyslipidemia and heart failure, and creatinine. To further examine these associations in individuals with and without AF, the model using the second set of adjusting variables but without creatinine was expanded to include AF and an interaction between AF presence and homoarginine.

Multivariable logistic regressions for AF were performed with homoarginine as regressor. These models used the same three sets of adjusting variables used in the linear regressions and additionally the variables in (2) without creatinine but with the addition of heart rate.

For further age- and sex-adjusted linear regression analyses, we divided the study sample into the following subgroups:

1. Healthy controls with no cardiovascular risk factors (*N* = 689);
2. Individuals with at least one cardiovascular risk factor but without AF (*N* = 2943);
3. Participants with AF (*N* = 124).

## 3. Results

### 3.1. Baseline Data

In Table 1, the baseline characteristics for the total study sample and the AF group are presented.

Electrocardiographic and echocardiographic variables are shown in Table 2.

In patients with current sinus rhythm, the median PQ-interval was 158 ms and P wave duration was 110 ms in the total sample as well as in patients with no atrial fibrillation. In the subgroup with AF in history and current sinus rhythm (*N* = 74), the median PQ-interval was 168 ms (*p*-value = 0.0014) and P wave duration was 118 ms (*p*-value < 0.001).

The E/A ratio amounted to 1.1 in the total sample and both subgroups (*p*-value = 0.47).

### 3.2. Homoarginine, Electrocardiographic and Echocardiographic Variables

As shown in Table 3, there were no strong and statistically significant associations between plasma homoarginine and electrocardiographic variables (PQ interval, P wave duration, ventricular rate, QRS duration, QTc interval) in multivariable-adjusted linear regression analyses. Several echocardiographic variables were significantly related to homoarginine.

Models with adjustment for age and sex, and with risk factor adjustment including creatinine are provided in Supplementary Tables S1 and S2.

Analyses of homoarginine in relation to intermediate phenotypes in subgroups by AF status did not show marked differences (Supplementary Table S3). No significant interactions by AF status were observed.

### 3.3. Homoarginine and Atrial Fibrillation

**Figure 1:**
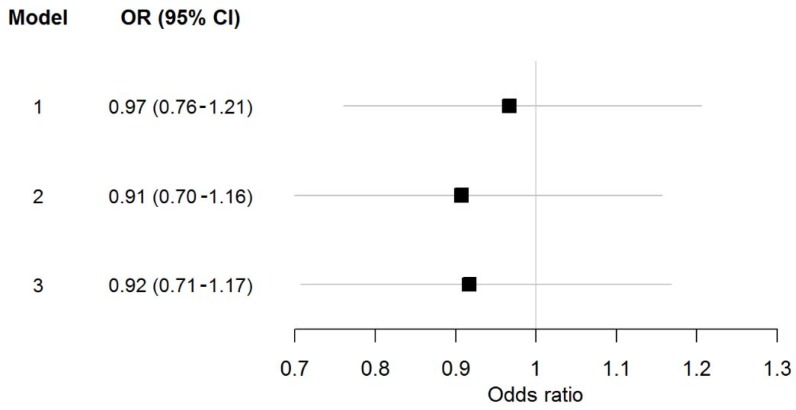
Multivariable-adjusted odd ratios for homoarginine per standard deviation increase in relation to atrial fibrillation (AF). Models: 1. Age- and sex-adjusted; 2. Risk factor-adjusted model including age, sex, body mass index, systolic blood pressure, antihypertensive medication, diabetes, active smoking, history of myocardial infarction, dyslipidemia, heart failure, and log creatinine; 3. Risk factor-adjusted model including age, sex, body mass index, systolic blood pressure, antihypertensive medication, diabetes, active smoking, history of myocardial infarction, dyslipidemia, heart failure, and heart rate. OR stands for odd ratios; CI for confidence interval.

In Figure 1, the data of multivariable-adjusted regression analysis for homoarginine in relation to atrial fibrillation is shown.

**Figure 2:**
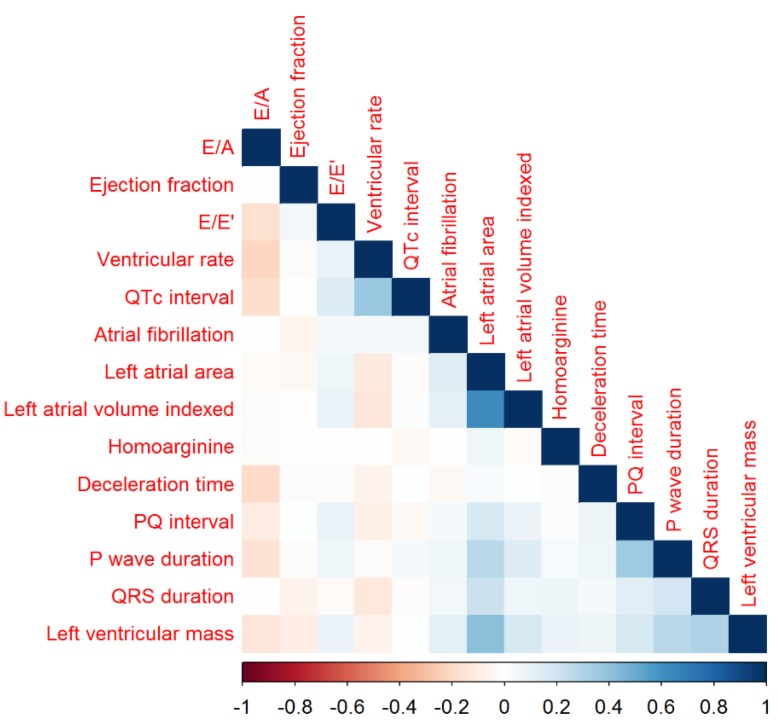
Kendall rank correlations between homoarginine, electrocardiographic and echocardiographic parameters, and atrial fibrillation (AF).

Kendall rank correlations between homoarginine, electrocardiographic and echocardiographic parameters, and atrial fibrillation are shown in Figure 2.

The linear regression analyses of the divided subgroups showed 0.1 higher homoarginine levels in patients with at least one cardiovascular risk factor but without AF (CI 0.03–0.17; *p*-value = 0.073) compared to healthy controls. Participants with AF presented 0.06 higher homoarginine levels (CI −0.10–0.22; *p*-value = 0.47).

## 4. Discussion

In our population-based cohort we can demonstrate an association of plasma homoarginine levels with left atrial size and diastolic mitral valve flow. No statistically significant relations with electrocardiographic variables or AF itself could be shown.

### 4.1. Homoarginine and Electrocardiographic and Echocardiographic Variables

We hypothesized that homoarginine may be related to electrocardiographic and echocardiographic parameters of atrial and ventricular conduction. In our population-based study, however, ventricular rate and QTc lost statistical significance of the association after careful adjustment for cardiovascular risk factors. The initial association of homoarginine with electrocardiographic variables may have been driven by cardiovascular risk factors and disappeared after they entered the equation. These findings are inconsistent with a previous study in chest pain unit patients which demonstrated an association between homoarginine, ventricular rate, and QTc [25].

In our study, homoarginine showed statistically significant associations with echocardiographic variables of left atrial size and the E/A ratio although they were weak. We also observed a relation of homoarginine and left ventricular mass. All three variables have been related to cardiovascular risk and AF [27,28,29,30,31,32,33,34].

Left atrial size as an indicator of left atrial remodeling and diastolic dysfunction is a strong indicator of cardiovascular morbidity and AF [27,28,29,30,31]. Furthermore, E/A ratio is also known as a marker for diastolic dysfunction [32]. Therefore, our findings indicate that homoarginine could be an additional marker for diastolic dysfunction in the general population. Our observations are consistent with a previous study in a subgroup of high risk individuals for diastolic heart failure that showed a statistically significant association of homoarginine with echocardiographically determined diastolic function and N-terminal pro B-type natriuretic peptide [35].

Similarly, lower plasma homoarginine was also related to ventricular mass in the study participants [35]. An elevated left ventricular mass expresses left ventricular hypertrophy, which is a well-known prognostic factor of cardiovascular morbidity and mortality [33,34]. Therefore, our findings, although they reached only borderline significance, are in line with the results of Drechsler et al. who could demonstrate a strong correlation of low homoarginine concentrations with prevalence of left ventricular hypertrophy as well as congestive heart failure and high levels of brain natriuretic peptide in patients with diabetes under haemodialysis [36].

Atzler et al. described that a four-week dietary supplementation with homoarginine led to significant positive effects on left ventricular contractility and diastolic function [37]. Our data cannot confirm the previous findings of an association of homoarginine levels and left ventricular ejection fraction [20]. Overall, the associations with echocardiographic variables of left ventricular structure and diastolic function are in line with recent publications that support low homoarginine as a marker for mortality and cardiovascular disease, in particular heart failure [16,18,20,36]. Due to the lack of a strong association between homoarginine and left ventricular ejection fraction, our results suggest a more specific relation with diastolic heart failure and normal ejection fraction which must be proven in functional studies.

### 4.2. Homoarginine and Atrial Fibrillation

Our findings suggest that there is no strong clinical association of homoarginine with AF that would render it suitable as a novel biomarker for AF. Whether it is useful to improve our understanding of the pathophysiology of AF cannot be shown by our data.

Oxidative stress measured by reactive oxidative metabolites is correlated with cardiac conduction disturbances [38]. Nitric oxide (NO) is central for cardiovascular homeostasis and autonomic control [39,40] and has been reported to be related to heart rate [41,42]. Although recent studies suggested that arginase inhibition is unlikely to play a significant role in the reported cardio-protective effects of homoarginine [43], it has been shown that the imbalance of beneficial L-arginine derivatives such as asymmetric dimethylarginine and homoarginine results in oxidative and nitrosative stress [44].

Endothelial function and asymmetric dimethylarginine induced oxidative stress have been reported in context with new onset AF in acute myocardial infarction [44]. It has also been shown that prevalent and incident AF are associated with increased oxidative stress levels, which was measured by the redox potentials of glutathione [45]. Xie et al. showed an increasing prevalence of AF with higher oxidative stress levels and identified a link between oxidative stress and aberrant intracellular Ca^2+^ release via the type 2 ryanodine receptor (RyR2). In this study genetic inhibition of mitochondrial reactive oxygen species production and pharmacological treatment of RyR2 leakage prevented AF [46]. In contrast, recent research tested the effect of an oral anti-oxidant treatment (α-lipoic acid) on AF recurrence in patients after catheter ablation. In this investigation, α-lipoic acid therapy reduced only serum levels of common markers of oxidative stress but did not prevent AF recurrence after an ablative treatment [47].

In addition, cardiac remodeling also seems to have a role in the development of AF and vice versa. This is supported by a study which showed a prevention of new-onset atrial fibrillation in patients with non-ischemic dilated cardiomyopathy and a positive response to cardiac resynchronization therapy due to less reverse left ventricular remodeling [48,49].

Despite the role of homoarginine in the NO pathway, there does not appear to be a clinically relevant association of circulating homoarginine with AF in individuals from the general population. These findings are in line with recent studies that demonstrated that other biomarkers of oxidative stress such as glutathione-peroxidase-1, myeloperoxidase, and asymmetric dimethylarginine were not associated strongly with prevalent AF after adjustment for cardiovascular risk factors [50,51].

The results in ambulatory individuals from the community could not validate previous observational studies, which reported an association between low homoarginine levels and AF prevalence. In acute chest pain patients, homoarginine was correlated with biomarkers of myocardial stress such as B-type natriuretic peptide and coronary ischemia including high sensitivity measured troponin I [25]. Homoarginine was measured lower in individuals with AF and was predictive of adverse outcomes. In a study in oesophagectomy patients, homoarginine was related to postoperative AF [26]. These cohorts comprised patients of high cardiovascular risk and were characterized by acute states of inflammation and cardiac stress, which may explain the clear association observed in a different setting. The association of homoarginine to AF in the previous findings may also be mediated by an enlarged left atrium, which increases the risk of AF occurrence, recurrence, and persistence.

We also showed that individuals with a high burden of cardiovascular risk factors tended to have higher homoarginine levels. This matches previous findings where homoarginine was related to smoking, hypertension, and metabolic disease as well as multiple cardiovascular diseases [17,18,52].

In our research, despite the higher prevalence of cardiovascular risk factors in the AF group, we could not verify the thesis of other literature, which indicated lower homoarginine levels in patients with cardiovascular disease. This may be due to the low sample size of participants with AF in our study or due to better treatment of cardiovascular risk factors in patients with diagnosed AF. To investigate this paradox, further research will be needed.

However, our findings suggest that homoarginine might be relevant in patients with left ventricular hypertrophy and atrial fibrillation. For further investigation of the pathophysiological aspects of AF and a potential targeted therapeutic approach, additional research is needed.

### 4.3. Limitations and Strengths

In our study we present data on prevalent AF which may dilute temporal relations and results may be affected by reverse causation. The number of cases was comparatively low and may have limited our power. The confidence intervals are wide, respectively. However, strong, clinically relevant associations of homoarginine and AF should have been detected. Since our study lacks long-term monitoring for AF we may have missed paroxysmal, asymptomatic cases. This misclassification may have biased our results towards the null.

The assessment of diastolic function was limited by the lack of availability of echocardiographic markers to properly classify diastolic impairment. In particular, the use of continuous variables such as E/A does not provide the full picture of diastolic performance. Results should be interpreted with caution.

Random variation may have affected our results. Furthermore, we cannot exclude residual confounding that may have masked associations despite rigorous adjustment for possibly relevant covariates.

Nevertheless, we present results in a contemporary, well-defined population-based sample with interdisciplinary case adjudication and standardized assessment of clinical variables. Our data may thus guide future research.

## 5. Conclusions

In summary, we could not identify statistically significant correlations between homoarginine levels and AF nor with most electrocardiographic phenotypes. Homoarginine may not represent a strong biomarker to identify individuals at increased risk for AF. However, we found associations between homoarginine and echocardiographic intermediate phenotypes. Further investigations will be needed to elucidate the role of homoarginine and cardiac function.

## Figures and Tables

**Table 1:** Characteristics of the study cohort and in the subgroups by atrial fibrillation (AF) status.

	Total Sample (*N* = 3761)	No Atrial Fibrillation (*N* = 3606)	Atrial Fibrillation (*N* = 124)	*p*-Value
Age (years)	56.0 (46.0, 65.0)	55.0 (46.0, 64.0)	67.0 (60.0, 72.0)	<0.001
Males, *N* (%)	1946 (51.7)	1836 (50.9)	91 (73.4)	<0.001
Heart rate (bpm)	68.0 (61.5, 76.0)	68.0 (61.5, 75.5)	69.0 (59.5, 79.0)	0.72
**(a) Cardiovascular risk factors and diseases**				
Body mass index (kg/m^2^)	26.6 (24.0, 29.8)	26.5 (23.9, 29.7)	28.2 (25.3, 32.3)	<0.001
Active smoker, *N* (%)	693 (18.5)	675 (18.8)	13 (10.5)	0.027
Diabetes, *N* (%)	290 (7.7)	272 (7.5)	16 (12.9)	0.043
Dyslipidemia, *N* (%)	1059 (28.2)	1003 (27.8)	47 (36.7)	0.019
Family history of myocardial infarction, number (%)	678 (18.0)	647 (17.9)	25 (20.2)	0.61
Hypertension, *N* (%)	1952 (51.9)	1846 (51.2)	87 (70.2)	<0.001
Heart failure, *N* (%)	754 (20.1)	682 (19.0)	61 (49.2)	<0.001
**(b) Biomarkers**				
Homoarginine (µmol/L)	2.0 (1.5, 2.5)	2.0 (1.5, 2.5)	1.9 (1.5, 2.5)	0.56
Creatinine (mg/dL)	0.9 (0.8, 1.0)	0.9 (0.8, 1.0)	0.9 (0.8, 1.1)	<0.001

For continuous variables, median (25th percentile, 75th percentile) are given, and for binary variables, absolute and relative frequencies are provided. Bpm stands for beats per minute. The *p*-value given is for the Mann–Whitney (*U*) test for continuous variables and the chi-squared test (χ^2^) for binary variables.

**Table 2:** Electrocardiographic and echocardiographic clinical variables by AF status.

	Total Sample (*N* = 3761)	No Atrial Fibrillation (*N* = 3606)	Atrial Fibrillation (*N* = 124)	*p*-Value
**(a) Electrocardiographic variables**				
Ventricular heart rate (bpm)	61 (55, 67)	61 (55, 67)	65 (54, 77)	<0.001
QRS duration (msec)	94 (88, 102)	94 (88, 102)	96 (92, 109)	<0.001
QTc interval (msec)	420 (404, 435)	419 (404, 435)	430 (407, 450)	<0.001
**(b) Echocardiographic variables**				
Left atrial area (cm^2^)	18 (16, 21)	18 (16, 21)	23 (19, 28)	<0.001
E/E’	7.0 (5.8, 8.7)	7.0 (5.7, 8.7)	7.8 (6.4, 9.8)	<0.001
Deceleration time (msec)	225 (194, 260)	225 (194, 260)	213 (173, 257)	0.0084
Left ventricular ejection fraction (%)	64. (60, 68)	64 (60, 68)	61 (57, 68)	<0.001
Left ventricular mass (g)	153 (125, 185)	152 (123, 183)	202 (157, 241)	<0.001
Left atrial area indexed by body surface area (cm^2^/m^2^)	10 (9, 11)	10 (9, 11)	11 (10, 14)	<0.001

For continuous variables, median (25th percentile, 75th percentile) are given, and for binary variables, absolute and relative frequencies are provided. The *p*-value given is for the Mann–Whitney test for continuous variables and the chi-squared test for binary variables.

**Table 3:** Multivariable-adjusted linear regression analyses of homoarginine in relation to electrocardiographic and echocardiographic variables in the total cohort.

Clinical Variables	Beta (95% CI)	Beta per SD (95% CI)	*p* Value
PQ interval	−0.24 (−1.14, 0.66)	−0.20 (−0.96, 0.55)	0.6
P wave duration	−0.38 (−0.86, 0.10)	−0.32 (−0.72, 0.08)	0.12
Ventricular rate	0.30 (−0.09, 0.69)	0.25 (−0.08, 0.58)	0.14
QRS duration	−0.10 (−0.60, 0.40)	−0.08 (−0.50, 0.34)	0.7
QTc interval	0.35 (−0.53, 1.24)	0.30 (−0.45, 1.05)	0.43
Left atrial area	−0.15 (−0.28, −0.02)	−0.12 (−0.23, −0.02)	0.024
E/A	−0.01 (−0.03, −0.003)	−0.01 (−0.02, −0.003)	0.013
E/E’	0.05 (−0.05, 0.15)	0.04 (−0.04, 0.12)	0.3
Deceleration time	0.76 (−1.24, 2.75)	0.64 (−1.05, 2.33)	0.46
Left ventricular ejection fraction	0.19 (−0.06, 0.44)	0.16 (−0.05, 0.37)	0.13
Left ventricular mass	−1.44 (−2.88, 0.00)	−1.21 (−2.43, 0.004)	0.051

CI: confidence interval; SD: Standard deviation.

## Supplementary Materials

The following are available online at http://www.mdpi.com/2218-273X/8/3/86/s1, Table S1: Linear regression analyses for homoarginine in relation to electrocardiographic, and echocardiographic variables in the total cohort, adjusted for age and sex, Table S2: Multivariable-adjusted linear regression analyses of homoarginine in relation to electrocardiographic, and echocardiographic variables in the total cohort, adjusted for cardiovascular risk factors and creatinine, Table S3: Multivariable-adjusted linear regression analyses of homoarginine in relation to electrocardiographic and echocardiographic variables including the interaction of homoarginine with AF status [53].
