# Supplementary material for: Cross-Sectional Associations between Homoarginine, Intermediate Phenotypes, and Atrial Fibrillation in the Community—The Gutenberg Health Study
Source: Biomolecules. 2018 Aug 30;8(3):86. doi: 10.3390/biom8030086 (PMC6165554; doi:10.3390/biom8030086)
Supplement: Supplementary file 1 [file biomolecules-08-00086-s001.pdf]

biomolecules

MDPI

Table S1. Linear regression analyses for homoarginine in relation to electrocardiographic, and echocardiographic variables in the total cohort, adjusted for age and sex.

Clinical Variables	Beta (95% CI)	Beta per SD (95% CI)	*P*-Value
PQ interval	0.49 (−0.40–1.38)	0.41 (−0.34–1.16)	0.28
P wave duration	0.18 (−0.30–0.66)	0.15 (−0.25–0.56)	0.46
Ventricular rate	0.55 (0.15–0.95)	0.47 (0.13–0.81)	0.0069
QRS duration	0.06 (−0.44–0.55)	0.05 (−0.37–0.47)	0.82
QTc interval	1.02 (0.12–1.92)	0.86 (0.10–1.62)	0.026
Left atrial area	0.09 (−0.05–0.23)	0.08 (−0.04–0.19)	0.19
E/A	−0.03 (−0.04–(−0.01))	−0.02 (−0.03–(−0.01))	<0.001
E/E'	0.11 (0.01–0.21)	0.10 (0.01–0.18)	0.025
Deceleration time	0.65 (−1.32–2.61)	0.55 (−1.11–2.21)	0.52
Left ventricular ejection fraction	0.29 (0.02–0.55)	0.24 (0.02–0.46)	0.032
Left ventricular mass	1.31 (−0.25–2.88)	1.11 (−0.21–2.43)	0.099

Table S2. Multivariable-adjusted linear regression analyses of homoarginine in relation to electrocardiographic, and echocardiographic variables in the total cohort, adjusted for cardiovascular risk factors and creatinine.

Clinical Variables	Beta (95% CI)	Beta per SD (95% CI)	*P*-Value
PQ interval	−0.24 (−1.14–0.66)	−0.20 (−0.96–0.56)	0.60
P wave duration	−0.37 (−0.85–0.11)	−0.31 (−0.72–0.09)	0.13
Ventricular rate	0.29 (−0.10–0.68)	0.24 (−0.09–0.57)	0.15
QRS duration	−0.10 (−0.60–0.40)	−0.08 (−0.50–0.34)	0.70
QTc interval	0.35 (−0.54–1.24)	0.29 (−0.46–1.04)	0.45
Left atrial area	−0.15 (−0.28–(−0.02))	−0.13 (−0.23–(−0.02))	0.021
E/A	−0.01 (−0.03–(−0.003))	−0.01 (−0.02–(−0.002))	0.014
E/E'	0.05 (−0.05–0.15)	0.04 (−0.04–0.12)	0.31
Deceleration time	0.76 (−1.23–2.76)	0.64 (−1.04–2.33)	0.45
Left ventricular ejection fraction	0.18 (−0.06–0.43)	0.16 (−0.05–0.36)	0.14
Left ventricular mass	−1.44 (−2.88–0.00)	−1.22 (−2.44–0.00)	0.05

Table S3. Multivariable-adjusted linear regression analyses of homoarginine in relation to electrocardiographic and echocardiographic variables including the interaction of homoarginine with AF status.

	Category	Beta (95% CI)	Beta per SD (95% CI)	*P* Value
	(a)	**Electrocardiographic variables**		

	Category	Beta (95% CI)	Beta per SD (95% CI)	*P* Value
		**PQ interval**		
*P*interaction 0.58				
	No AF	−0.2 (−1.11–0.7)	−0.17 (−0.93–0.59)	0.66
	AF	−2.03 (−8.42–4.36)	−1.71 (−7.11–3.68)	0.53
		**P wave duration**		
*P*interaction 0.095				
	No AF	−0.43 (−0.92–0.05)	−0.37 (−0.77–0.04)	0.076
	AF	2.48 (−0.91–5.87)	2.09 (−0.77–4.95)	0.15
		**Ventricular heart rate**		
*P*interaction 0.71				
	No AF	0.34 (−0.05–0.74)	0.29 (−0.05–0.62)	0.091
	AF	−0.05 (−2.06–1.96)	−0.04 (−1.74–1.66)	0.96
		**QRS duration**		
*P*interaction 0.080				
	No AF	0.01 (−0.49–0.52)	0.01 (−0.42–0.44)	0.96
	AF	−2.33 (−4.9–0.25)	−1.97 (−4.14–0.21)	0.076
		**QTc interval**		
*P*interaction 0.23				
	No AF	0.49 (−0.41–1.39)	0.41 (−0.35–1.17)	0.29
	AF	−2.35 (−6.94–2.24)	−1.99 (−5.86–1.89)	0.32
	(b)	**Echocardiographic variables**		
		**Left atrial area**		
*P*interaction 0.22				
	No AF	−0.13 (−0.26–0)	−0.11 (−0.22–0)	0.047
	AF	−0.55 (−1.2–0.11)	−0.46 −1.01–0.09)	0.10
		**E/A**		
*P*interaction 0.50				
	No AF	−0.01 (−0.03–0)	−0.01 (−0.02–0)	0.019
	AF	−0.04 (−0.12–0.04)	−0.03 (−0.1–0.03)	0.29
		**E/E'**		
*P*interaction 0.92				
	No AF	0.04 (−0.05–0.14)	0.04 (−0.05–0.12)	0.38
	AF	0.07 (−0.43–0.57)	0.06 (−0.36–0.48)	0.78
		**Deceleration time**		
*P*interaction 0.20				

	Category	Beta (95% CI)	Beta per SD (95% CI)	*P* Value
	No AF	0.4 (−1.62–2.43)	0.34 (−1.37–2.06)	0.70
	AF	7.21 (−3.08–17.5)	6.1 (−2.6–14.8)	0.17
**Left ventricular ejection fraction**				
*P*interaction 0.096				
	No AF	0.15 (−0.1–0.4)	0.13 (−0.08–0.34)	0.23
	AF	1.24 (−0.02–2.51)	1.05 (−0.02–2.12)	0.054
**Left ventricular mass**				
*P*interaction 0.21				
	No AF	−1.22 (−2.67–0.24)	−1.03 (−2.25–0.2)	0.10
	AF	−5.97 (−13.35–1.4)	−5.05 (−11.28–1.18)	0.11

SD: standard deviation, CI: Confidence interval. The regressors comprised homoarginine, atrial fibrillation, their interaction and cardiovascular risk factors including age, sex, body mass index, systolic blood pressure, antihypertensive medication, diabetes, active smoking, family history of myocardial infarction, dyslipidemia and congestive heart failure. E/A and E/E′ were available in *N* = 78 individuals with paroxysmal AF who were not in AF during the study ECG. For these analyses confidence intervals (and p-values) for the HOMO coefficient on each category (no AF or AF) are computed using the methods described in Figueiras, Domenech-Massons, and Cadarso (1998) [53].

References

53. Figueiras, A.; Domenech-Massons, J.M.; Cadarso, C. Regression models: calculating the confidence interval of effects in the presence of interactions. *Stat. Med.* **1998**, *17*, 2099–2105.

© 2018 by the authors. Submitted for possible open access publication under the terms and conditions of the Creative Commons Attribution (CC BY) license (http://creativecommons.org/licenses/by/4.0/).
